# Understanding Specific Contexts of Antiretroviral Therapy Adherence in Rural South Africa: A Thematic Analysis of Digital Stories from a Community with High HIV Prevalence

**DOI:** 10.1371/journal.pone.0148801

**Published:** 2016-02-29

**Authors:** Astrid Treffry-Goatley, Richard Lessells, Pam Sykes, Till Bärnighausen, Tulio de Oliveira, Relebohile Moletsane, Janet Seeley

**Affiliations:** 1 Africa Centre for Population Health, University of KwaZulu-Natal, Durban, KwaZulu-Natal, South Africa; 2 School of Education, University of KwaZulu-Natal, Durban, KwaZulu Natal, South Africa; 3 Department of Clinical Research, London School of Hygiene and Tropical Medicine, London, United Kingdom; 4 Independent digital storytelling practitioner and researcher, Digital storytelling South Africa, Cape Town, South Africa; 5 Department of Global Health and Population, Harvard T.H. Chan School of Public Health, Boston, Massachusetts, United States of America; 6 School of Laboratory Medicine and Medical Sciences, College of Health Sciences, University of KwaZulu-Natal, Durban, South Africa; 7 Honorary, Research Department of Infection, University College London (UCL), London, United Kingdom; 8 Research Department of Infection, University College London (UCL), London, United Kingdom; 9 Anthropology and Health, London School of Hygiene and Tropical Medicine, London, United Kingdom; University of Missouri-Kansas City, UNITED STATES

## Abstract

Near-perfect adherence to antiretroviral therapy (ART) is required to achieve the best possible prevention and treatment outcomes. Yet, there have been particular concerns about the challenges of adherence among patients living in resource-limited settings in sub-Saharan Africa. The primary objective of this study was to explore adherence in a low-resourced, rural community of high HIV prevalence in South Africa and to identify specific individual and structural factors that can either challenge or support adherence in this context. We applied digital stories as a qualitative research tool to gain insights into personal contexts of HIV and ART adherence. Through an inductive thematic analysis of twenty story texts, soundtracks and drawings, we explored experiences, understandings, and contexts of the participants and identified potential barriers and facilitators for those on lifelong treatment. We found that many of the stories reflected a growing confidence in the effectiveness of ART, which should be viewed as a key facilitator to successful adherence since this attitude can promote disclosure and boost access to social support. Nevertheless, stories also highlighted the complexity of the issues that individuals and households face as they deal with HIV and ART in this setting and it is clear that an overburdened local healthcare system has often struggled to meet the demands of a rapidly expanding epidemic and to provide the necessary medical and emotional support. Our analysis suggests several opportunities for further research and the design of novel health interventions to support optimal adherence. Firstly, future health promotion campaigns should encourage individuals to test together, or at least accompany each other for testing, to encourage social support from the outset. Additionally, home-based testing and ART club interventions might be recommended to make it easier for individuals to adhere to their treatment regimens and to provide a sense of support and solidarity.

## Introduction

### Opening story

My mother fell pregnant and went to the clinic. They tested her for HIV and found that she was infected. But, she never told anyone at home until the delivery. She gave birth at hospital and they tested her again. Again, they found her to have HIV. At the hospital, they advised her to tell at least one person at home in order for them to help her and remind her to take her pills properly. Since I was still young, I was 12 years old, my elder sister went to the hospital and they explained to her how mother should take her pills. […] When mother returned home, she called all of her children together. She told us what they had found at the hospital. My elder brother did not accept my mother’s status. The sister who went to learn for mother also did not support her. Therefore, mother did not take her pills. When I realised what she was doing, I saw that mother would die. I went to school and spoke to a teacher about the situation at home and mum’s antiretroviral therapy (ART). The teacher came home with me to meet mum. She explained that it is important to take her pills and not to bury them. Since that day, mother takes her pills correctly, and I support her. After sometime, my elder sister also got sick and found that she has HIV. In the end, my mother and I cared for her, even though she never supported mother. Today, they are *both well and they both take their ART*. *I care for both of them*. (Woman aged 20)

### Background

Near perfect adherence to antiretroviral therapy (ART) is required to achieve the best possible prevention and treatment outcomes. There have been particular concerns about the challenges of adherence to ART among patients living in resource-limited settings in sub-Saharan Africa [1; 2]. For example, our colleagues [3] have found that in the rural sub-district of Hlabisa, in KwaZulu-Natal province, adherence has proved to be a significant challenge to HIV treatment management, with around one in four adults having a suboptimal response to ART after one year on treatment. Moreover, results from a recent study in the same area, which implemented genotypic resistance testing, found that 80% of those with a poor response to ART had evidence of significant drug resistance [4; 5]. The development of drug resistance can be directly linked to inadequate adherence and, consequently, there is a critical need for research to understand better the nature of adherence in this and similar contexts.

Literature on ART adherence from different settings and contexts has indicated that both individual and structural factors play a role in ART adherence. Individual factors include disclosure [6; 7], substance abuse [8], stigma and depression [8; 9]. Structural barriers include: (i) poverty related issues, such as poor social support, a lack of transportation systems, food insecurity and the influence of social protection schemes such as disability grants or pensions; (ii) institutional factors like overburdened healthcare facilities, limited access to mental health services and inadequate counselling; (iii) political and cultural barriers including traditional beliefs about HIV, migration, poor health literacy and gender inequalities [10].

Since optimal adherence to ART is so important, numerous interventions have been designed to promote good adherence. However, for every study that showed one type of intervention to work, there was at least one other equally rigorous study that failed to find any effect, with the exception of mobile phone messages [11; 12]. One explanation for this finding is that the context in which an intervention is implemented is critical for intervention success. Therefore, there is a great need for further research to understand better specific contexts of ART adherence.

The story quoted above was recorded at a digital storytelling workshop held in a rural sub-district of Hlabisa. Digital storytelling is an emerging community based participatory research methodology that combines oral storytelling traditions with computer and video production technology. Over the past fifteen years, digital storytelling has been used in a variety of contexts, including education, health research, community engagement, violence prevention and social advocacy as a way to learn about society, from the local people’s perspective [13; 14]. However, to the best of our knowledge, digital storytelling has not yet been applied to explore community understandings of ART adherence in South Africa.

The primary goal of the study reported in this paper was to understand the nature of adherence in a low-resourced, rural community of high HIV prevalence in South Africa and to identify specific individual and structural factors that can either challenge or support adherence in this context. The insights gained through this analysis of community-authored digital stories could help to design and develop adherence-enhancing interventions for this particular population and the theoretical insights that we gain could be transferred to other settings and could potentially support intervention design in other places.

## Materials and Methods

### Setting

The Africa Centre for Population Health (Africa Centre) launched this three-year study in 2013. Africa Centre is an international research facility in KwaZulu-Natal, South Africa that is funded by the Wellcome Trust. It is located in the uMkhanyakude district, a rural area about two hours north of the largest provincial town of Durban, which is characterised by high levels of poverty. The total unemployment rate was 52.6% at the time of the 2011 Census, rising to 61.9% among youth. HIV prevalence in the area is high (29% in the adult population aged 15–49 years in 2011) [15]. HIV treatment and care has been delivered through a decentralised primary health care programme since 2004 [16]. By 2010, 40% of the population lived in a household with at least one member in HIV care and 25% in a household with at least one member on ART [17]. The scale-up of ART has had a profound impact on population-level mortality, with a rise in adult life expectancy of 11 years between 2003 and 2011 [18].

### Digital storytelling details

In this paper, we focus on digital stories as a qualitative research tool to show how community-authored accounts have the potential to provide valuable insights into personal contexts of HIV and ART adherence. Through an analysis of twenty digital stories, we aim to elucidate the experiences, understandings, and contexts of the participants and to identify potential barriers and facilitators for those on lifelong treatment in this under-resourced, rural community of HIV high prevalence. Below, we provide a detailed explanation of how we applied digital storytelling in this study.

We decided to apply digital storytelling as a methodology in this project, since it can be particularly relevant when working with minority and underserved communities and can encourage individuals to take part in research on local health issues [19]. Colleagues have applied a related methodology called Photovoice in their work with rural women to privilege ‘the voices of those mostly affected–in this case, the women–rather than those of the usual experts in identifying the issues and developing interventions to address them’ [20]. Indeed, it is only when ‘the most marginalized themselves are engaged in identifying the issues that affect them and the possible solutions for addressing them, that interventions are more likely to work’ [20].

#### Participant recruitment

Recruitment for this study took place over the first two weeks of October 2013. We advertised a workshop about ART called ‘Adherence Stories’ at local primary healthcare clinics (PHC) and recruited a convenience sample of attendees. For ethical reasons, we decided to make workshops open to all adults regardless of their HIV status and approached all the people in the PHC facility on a given day. We did not ask participants to disclose their HIV status at any point. The local community responded well to the project and we received a total of ninety-six applicants. Of these individuals, we selected twenty to attend the workshop.

#### Participant selection

The project team conducted the selection independently. The selection criteria included 1) motivation for attendance, and 2) an intention to include a balanced representation of age and gender. In terms of motivation, we gave preference to individuals who expressed a strong desire to share a story from their lives.

### Workshop details

We ran two digital storytelling workshops in November 2013. One workshop ran during the week and the second over two weekends so that both employed and unemployed people could attend. Ten individuals attended each workshop and twenty short films were produced. The workshops took place over four days with a break between the second and third day (kindly refer to Table 1 below).

**Table 1:** Workshop structure.

Workshop structure	
Day 1	Introduction, story circle (identify stories and share with the group) and photo tutorial
Day 2	Art tutorial, take photos and record stories.
Break	Facilitators created a draft of stories while participants produce further photos/ drawings at home.
Day 4	Produce music and generate second draft.
Day 5	Premiere of the films, discussion, provide final consent.

#### Day one

The facilitator introduced the concept of digital storytelling, showed relevant digital stories and explained the workshop process. The workshop was based on the ‘seven step’ process taught by the StoryCenter [13]. Once written consent had been secured, we invited participants to join in a story circle during which each person was given about twenty minutes to share their story with the group. After the story circle, we ran a short photographic tutorial to build participants’ ability and confidence to use their own photographs in their storytelling, both as direct illustration and as abstract metaphor; and secondly to inform them about techniques for making anonymous portraits, such as silhouettes or extreme close-ups. During the afternoon, we worked with participants individually to write up their stories into scripts of about 200–300 words.

#### Day two

While some facilitators gathered the photos taken the previous day, others laid out art materials and invited participants, under the guidance of a local art tutor, to create artworks to illustrate their stories. In a parallel process, we read through printed scripts privately with each participant to gain final approval before making an audio recording of each story. We used large pieces of cardboard to assemble excerpts of the printed stories with appropriate imagery such as drawings and printed photographs. Each participant was asked to complete their storyboard to the best of their ability and to think about additional pictures that might be needed.

#### Break

Participants were asked to take more photos, and to draw pictures at home. In the meantime, the facilitators prepared rough edits of the stories based on the storyboards, using the recorded voiceovers, photographs and artworks completed so far. It is important to note here that our process differed from that of a traditional digital storytelling workshop, during which participants are taught how to use video editing software so they can physically complete the creation of their own stories [13]. We decided that given the limited time available, the low literacy and lack of any computer skills among our participants, it would be more appropriate for the facilitators to do the physical editing, using the participants’ storyboards as instructions. We emphasized throughout that the participants were “creative directors” of their own stories and that that facilitators’ role was that of assistant.

#### Day three

We shared a first draft with each participant individually so that they could decide what they would like to change and improve. They identified visuals that they thought were missing and either filled in these gaps with additional photos or drawings, or by choosing one of the Africa Centre’s stock archive photos that were available for their use. We then ran a music workshop to create soundtracks for the stories.

#### Day four

On the last day of the workshop, after viewing the final products, participants were asked to sign a release form to confirm if and how they would like to share their stories. The screening was followed by an informal discussion and debriefing session.

### Data analysis

We applied narrative analysis techniques to interrogate the text, music, photographs and original artwork used in the digital stories. Narrative analysis ‘refers to a family of approaches’ in the human sciences ‘to diverse kinds of texts, which have in common a storied form’ [21]. We applied thematic analysis, which focuses on the meaning of the content and is ‘useful for theorising across a number of cases- finding common thematic elements across research participants and the events they report’ [21; 22]. In thematic analysis, investigators collect many stories and inductively create abstract categories from the data at hand. Accordingly, the story scripts were translated from Zulu into English and imported into the Nvivo Qualitative Data analysis software (Mac 10). We methodically familiarised ourselves with the information at hand and analysed the stories according to inductive thematic analytic procedures. Once familiar with the stories, two researchers independently coded the data in Nvivo. We each came up with themes based on repeated ideas that we identified in the text. We also attached a subjective statement as a subtitle for each theme to provide certain insight into some of the key meanings of these themes found in participant stories. These thematic categories were then crosschecked and reworked until consensus was drawn and we were satisfied with the organization of the data.

In terms of visual texts, for the sake of brevity and feasibility, we focused on the original drawings produced by the participants, rather than the stock images from the Africa Centre library that some participants used. Before data analysis proceeded, participant information was coded into root codes containing just the age and sex of each individual to ensure that the authors of the stories were not identifiable in published writings.

### Ethics

Ethics approval was obtained from the University of KwaZulu-Natal’s Biomedical Research Ethics Committee (BREC) in 2013 (2013–2015) (BE203/13). Written informed consent was obtained from all participants.

## Results

### Sample description

As mentioned above, ninety-six community members applied to attend the workshop. All of the applicants were black South African citizens or residents and just eight were men. In our interest to achieve a balanced gender ratio, we invited all eight of these individuals to attend the workshops. However, only one was able to come and so the participants consisted of nineteen women and one man. We had a relatively wide age range from twenty to fifty-three years, with a mean age of thirty years.

A total of twenty digital stories were produced at the workshops. Nineteen stories were told in native Zulu and one in English. Story length ranged from 1m35sec to 2m44s with an average length of 2m17sec. In Table 2 below, we have listed the codes of the stories, the English titles of the stories, the genre of the sound track song and the URL link for each story. All attendants provided full permission to share their stories with the public at the local clinics, with our research centre for educational purposes and online.

**Table 2:** Digital story details.

Code	English title	Genre of sound track	Figshare: Digital object identifier
Woman, 51	Arthritis	Christian choral	10.6084/m9.figshare.2069184
Woman, 35	Disclosure	Christian choral	10.6084/m9.figshare.2069187
Woman, 32	Mum was sick	Classical	10.6084/m9.figshare.2069190
Woman, 30	I was coughing	Christian choral	10.6084/m9.figshare.2069190
Woman, 28	Mum's illness	Classical	10.6084/m9.figshare.2069196
Man_21	Mum and granny	Classical	10.6084/m9.figshare.2069199
Woman, 53	Leave me virus!	Secular choral	10.6084/m9.figshare.2069202
Woman, 33	We don’t know everything	Christian choral	10.6084/m9.figshare.2069205
Woman, 27	Perseverance	Secular choral	10.6084/m9.figshare.2069208
Woman, 22	My parents	Classical	10.6084/m9.figshare.2069211
Woman, 29	Having HIV	Secular choral	10.6084/m9.figshare.2069214
Woman, 25	Mother and child	Christian choral	10.6084/m9.figshare.2069214
Woman, 25	Being uniformed	Indigenous	10.6084/m9.figshare.2069223
Woman, 22	My secret	Christian choral	10.6084/m9.figshare.2069226
Woman, 27	My mum and brother	Secular choral	10.6084/m9.figshare.2069229
Woman, 31	Danger	Indigenous song	10.6084/m9.figshare.2069232
Woman, 20	Mum and ART	Christian choral	10.6084/m9.figshare.2069235
Woman, 33	Accepting your status	Christian choral	10.6084/m9.figshare.2069238
Woman, 35	HIV and TB	Christian choral	10.6084/m9.figshare.2069241
Woman, 27	Pain in my life	Secular choral	10.6084/m9.figshare.2069244

### Story themes

A thematic analysis of narrative texts, triangulated with a visual and musical study of participant drawings and soundtracks, provided insight into participant experiences of HIV and ART adherence in this context and revealed six overlapping and nonexclusive themes that are outlined in Table 3 below.

**Table 3:** Story themes.

No:	Theme titles	Subtheme
**1**	Adherence: *Treatment is for life*	Armed struggle: *I am armed now and I am not going back!*
**2**	Illness and death: *The black nation is dying*	Transmission routes: *Hey HIV!* *Where did you come from?*
**3**	Health seeking behaviour and experience: *I am sick*, *I need help*	HIV testing: *Facing the fear*
**4**	Stigma: *Beware! She might infect us*	None
**5**	Disclosure: *Disclosure is not easy*	None
**6**	Social support: *We help each other*	None

### Adherence: Treatment is for life

Fifteen of the stories addressed the general topic of adherence, eleven of which were specifically about ART adherence. There were also stories about adherence to medications for other chronic disease, including tuberculosis (TB), arthritis, hypertension and asthma. A number of the stories alluded to a similarity between HIV and other chronic illnesses. For example: ‘I learned that most of the diseases are the same in life because like others, they need you to take treatment for the rest of your life’ (woman, aged 51).

#### Armed struggle: I am armed now and I am not going back!

**Fig 1:**
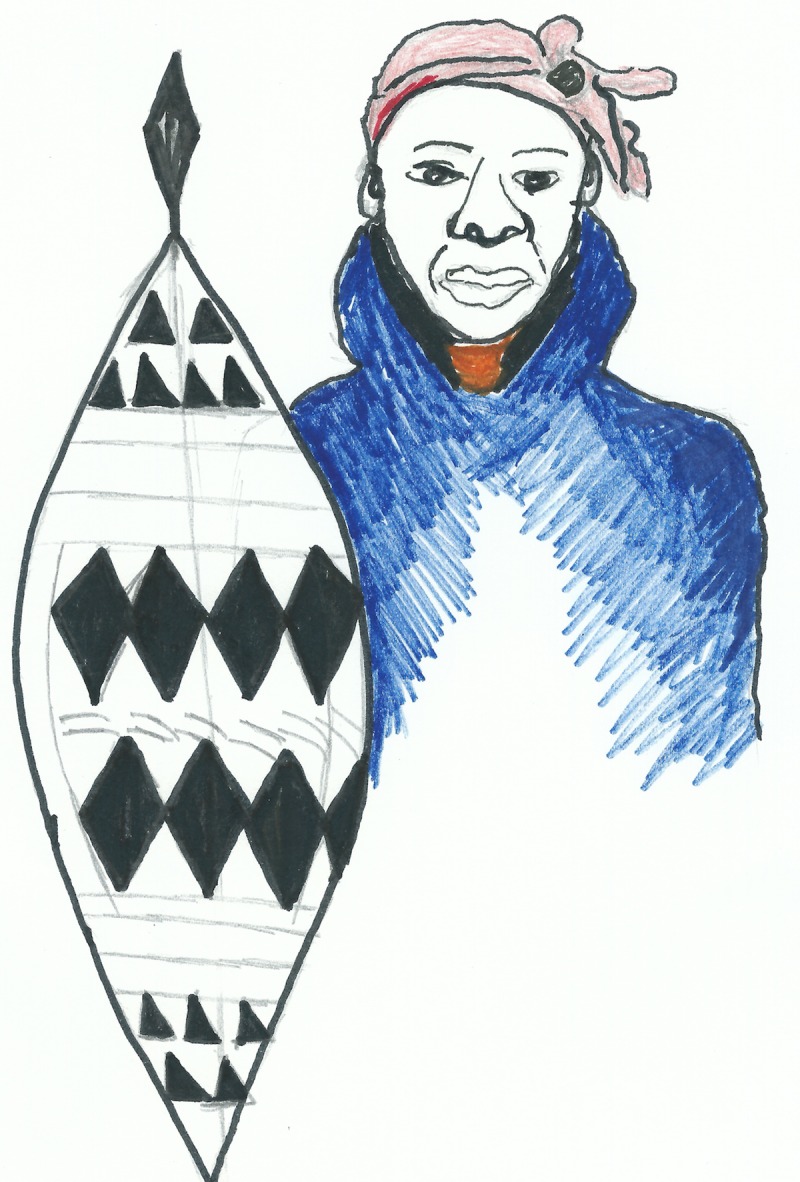
Participant drawing depicting the ‘armour’ of ART.

A subtheme of armed struggle emerged under the broader theme of adherence. For example, in the following excerpt ART is described as weaponry to protect an individual against HIV: ‘I took responsibility when the time came for me to take treatment. When I came back with my treatment, I told the virus that it could never defeat me. I also told myself that I am armed now and I am not going back’ (woman, aged 53). In a self-portrait drawn by the storyteller (Fig 1), she appears armed with a traditional Zulu shield.

The ‘armed struggle’ subtheme was also evident in an adaptation of the song ‘*Safa saphel’ isizwe’* (The black nation is dying) (written by Mbongeni Ngema), which was selected by three of the storytellers as a soundtrack. The original version of this song was used in the anti-apartheid film *Sarafina!*, directed by Darrel James Roodt (1992).

### Illness and death: The black nation is dying

All of the stories are about illness, and eleven of the twenty describe the death of one or more family members. Most of the stories speak about more than one illness, including cervical cancer, asthma, arthritis, hypertension, TB and HIV. Five of the stories describe personal experience of HIV and ten describe HIV in the family; five of these are about the storyteller’s HIV positive mother.

#### Transmission routes: Hey HIV! Where did you come from?

Nine of the stories about HIV deal directly with HIV transmission. Four allude to horizontal transmission, two of which link promiscuous behaviour and alcohol abuse to HIV infection. For example: ‘One day, my grandmother told me what kind of person mom was. She said that she liked boys and to drink alcohol. This led me to me to suspect that she was killed by this disease [HIV] that has spread’ (woman, aged 25).

The song *Hayi we ingculazi!* (Hey HIV!), chosen as a soundtrack by one storyteller, specifically addresses the issue of sexual transmission of HIV. The lyrics are quoted below for clarification:

*Hayi we Ingculazi!* (Hey! HIV)

*Hayi we ingculazi*, *wangena kanjalo?* (Hey HIV, how did you enter?)

*Wangena ngucansi*, *wangena kanjalo* (You entered through sex that is how you entered)

**Fig 2:**
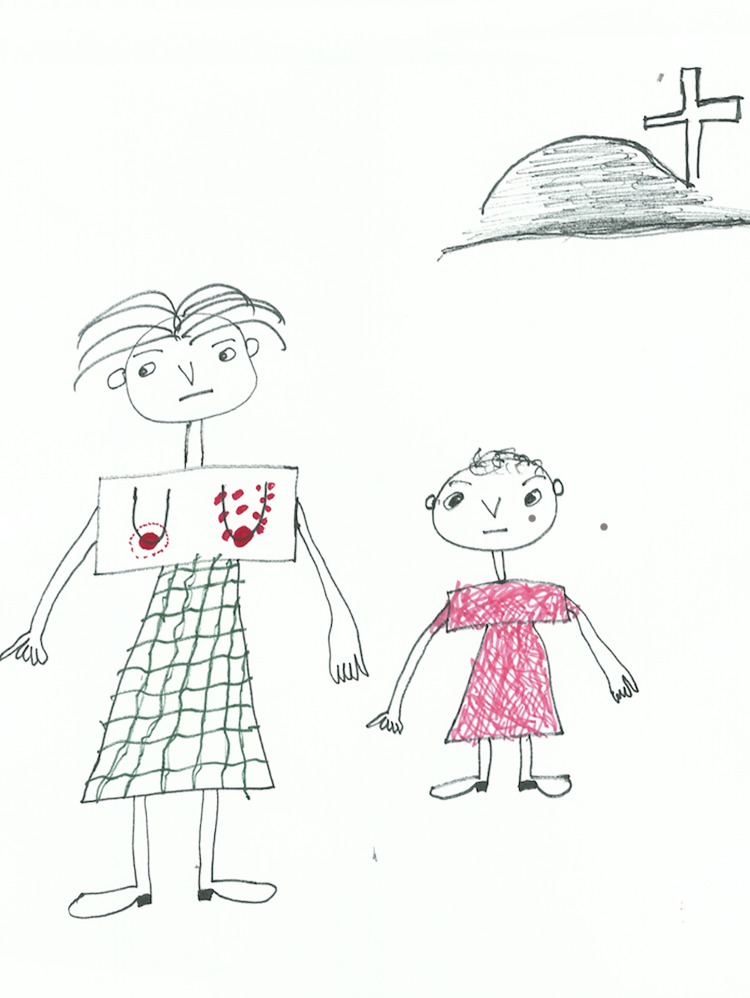
Participant drawing depicting the loss of a child.

Five stories are about vertical transmission: two describe successful prevention of mother to child transmission (PMTCT) and three describe mother to child transmission (MTCT). In one of the PMTCT stories, the storyteller proudly noted that ‘the baby is alive, schooling is not bothered by any sickness. My sister is alive, beautiful, taking her ART properly and the whole family is supporting her’ (woman, aged 25). In one of the MTCT cases, a child’s life is successfully extended through ART and ‘she can go to school because the treatment is serving her well’ (woman, aged 22). However, there were also two stories of children dying from HIV-related illness. Below is an account about a woman who transmitted HIV to her child. Fig 2 shown below accompanied this story.

In 1995, my elder sister got a baby. She was breastfeeding, but every time when breastfeeding, she was complaining about breast pains. Then, the breast had sores on its nipples, but she continued with breastfeeding … the baby started to get sick, vomiting and running stomach. After two years the baby died having sores in the mouth. (Woman, aged 25)

### Health seeking behaviour and experience: I am sick, I need help

Fourteen of the stories about HIV speak about starting ART, including the fear of possible side effects and experiences of medical plurality. For example:

My mother said I should wait a bit [before starting treatment]. She told me there is traditional concoction that she will look for. Then, she told one of the nurses we worship with at church. The Sister [nurse] spoke to me and told me it is necessary to start treatment because the concoction does both; it increases the CD4 count and the levels of the virus. When I started taking pills, I was scared because I heard that they change the body. When I took them I went to check in the mirror if they have changed me anywhere. (Woman, aged 29)

Four stories mention faith or traditional healing. These stories, including the one quoted above, all describe a certain tension between Western medicine (in this case antiretroviral drugs) and alternative approaches to healing. For example, in one story, the storyteller describes how her sick brother refused to seek medical help because he believed in traditional medicine. When she finally convinced him to go the clinic it was too late and he died (Woman, aged 35).

Fourteen of the stories describe seeking medical care from local primary health care clinics. Those who sought care for HIV related issues report mixed experiences. One storyteller recounted a painful story in which she and her sister went repeatedly and unsuccessfully to seek treatment for the same problem, only discovering the cause after the sister’s death. Two other stories describe repeated visits to the doctor with same problem. For example:

I started coughing, sweating and saw myself losing weight. I went to the doctor and he gave me medicine but I did not improve. When went to the doctor for the third time he told me to test for HIV. I agreed, but it came out negative. After that, I got abscesses all over my body. I spoke to a nurse who is also my neighbour. When I explained my problem she said that I have all the symptoms of HIV. Then, I went to the clinic to check for HIV. They found that I was infected. (Woman, aged 53)

#### HIV testing: Facing the fear

**Fig 3:**
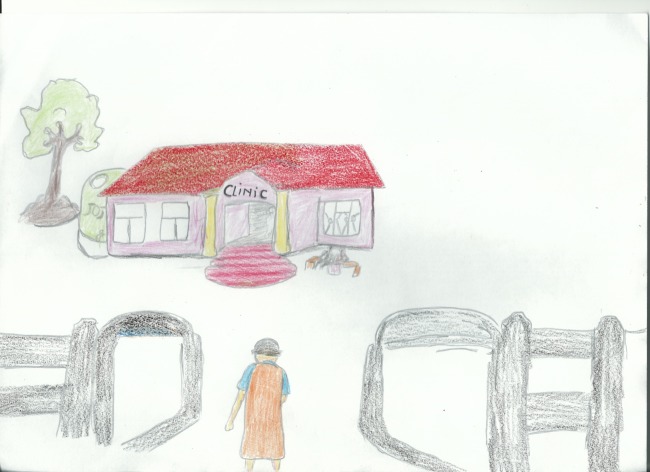
Participant drawing depicting walking to clinic to do an HIV test.

HIV testing is a clear subtheme under the health seeking behaviour and experiences theme, with thirteen stories explicitly mentioning going for an HIV test. Many participants chose to use stock pictures of local clinics to illustrate their journey to learn their status; others chose to draw their journey (see Fig 3 below).

While nine of the storytellers fell ill before going for the test, three tested when they fell pregnant and one did a home test with a local charity. One of the stories mentions fear of testing and two highlight feelings of sadness and depression following an HIV positive diagnosis. For example:

I had been telling myself all along that should I test positive, I would kill myself, but they made to agree to test. I was diagnosed HIV positive. My heart was sore, I cried, but they counselled me at the clinic that this was not the end of life. I went back and told my family, but I never told my brother. (Woman, aged 35)

#### 3.6. Stigma: *Beware! She might infect us*

**Fig 4:**
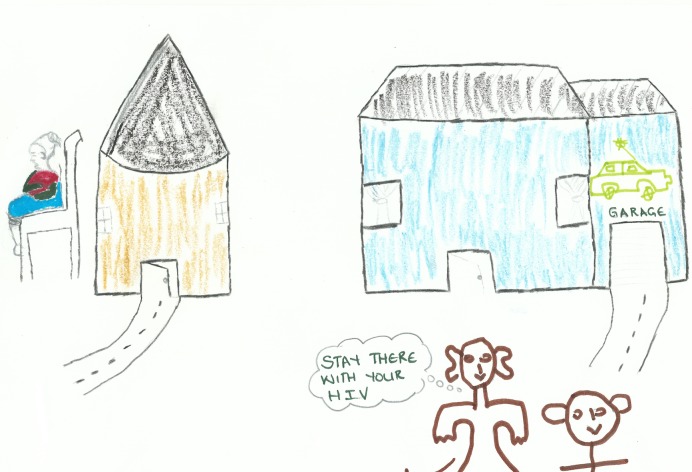
Participant drawing depicting the isolation of HIV stigma.

These accounts suggest that there is still stigma associated with HIV infection. This is evident in the numerous accounts of dishonesty about HIV-related death and illness, of fear of testing and of individuals who failed to disclose their HIV status to their loved ones. The stories also suggest that stigma, disclosure and social support are closely interwoven since a fear of stigma can have a major impact on a person’s ability to disclose and to receive the support that is key to successful adherence. For example, in the story quoted at the beginning of the paper, a mother refused to take her pills due to the discrimination that she experienced at the hands of her children. Another story, entitled ‘The Danger of Stigma’ illustrated with Fig 4 below, also showed how stigma can lead to isolation and division.

Although there is evidence in these stories of the negative impact of stigma in this community, there were also examples of individuals who firmly believed in their medications and adhered well in the face of discrimination. For example, the extract below provides an example of good adherence and resilience in the face of stigma.

All my aunties were laughing at me asking what I was studying for. But I continued taking my treatment. I ignored what they were saying about me. I eventually finished my TB treatment. It was difficult but I persevered because the nurse told me that if I was to live I must take my pills and I would recover. I was also thinking that my children would suffer without me. (Woman, aged 27)

### Disclosure: Disclosure is not easy

**Fig 5:**
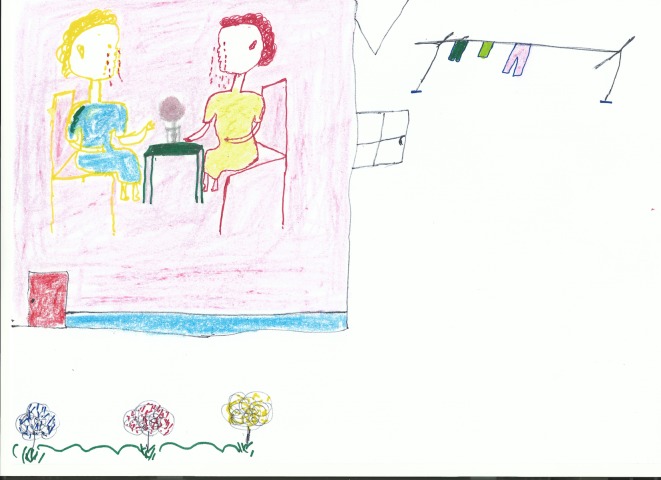
Participant drawing depicting the pain of disclosure.

In eleven of the stories the teller speaks about the process of disclosing their status to either their partner (two) or their family (nine). In the stories, lay HIV counsellors often recommended disclosure. For example: ‘Because of the counselling I received at the clinic, I saw that it better to disclose my status to my family’ (woman, aged 33). It is clear that disclosing one’s status is not an easy process. For example, in Fig 5 below, one can see the tears as a daughter discloses her status to her mother in the privacy of their home.

One story also revealed the violence that a woman encountered when she disclosed to her partner and others described how stigma could lead to secrecy, which can have a negative impact on disclosure. For example, a number of the storytellers expressed frustration over the failure of their elders to disclose and tell the truth about HIV. The following excerpt shows lack of honesty surrounding the status of the teller’s late sister’s surviving child:

My heart is sore. I do not get answers about my parent’s death. Also members of my family did not tell me the cause of their death. But they say it was TB. Now, I found that my late sister’s child has HIV. That made me notice that the virus has been here for very long and treatment was not available at the time. That gave me questions. Where did the child get the virus? I now have the answers. It got it from her mother. She did not die from TB. I feel bad as the child does not know what is wrong or why she takes treatment. (Woman, aged 22)

### Social support: We help each other

A number of narratives suggest a link between disclosure and receiving social support. For example, one storyteller explains how upon learning that she was HIV positive she disclosed her status to those at home. However, she failed to tell her brother. Sometime later, her brother fell ill but refused to test until she disclosed her status to him. They then went to the clinic together for him to test his status, but by the time he was diagnosed with HIV, it was too late for him (Woman, aged 35).

There were stories about family and community members supporting HIV positive people by reminding them to take their treatment. For example:

Until today, I live well without problems. I take my pills correctly. I thank the nurse who encouraged me to go for a test. She still supports me and even my family support me. My children, husband and neighbour support me by reminding me to take my treatment. (Woman, aged 53)

**Fig 6:**
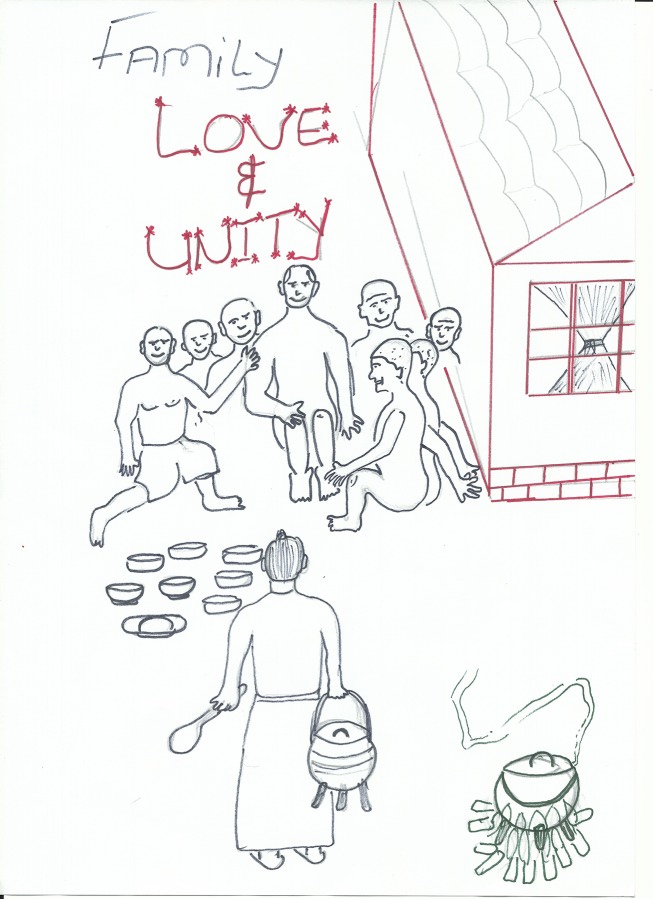
Participant drawing depicting the strength of family unity.

Other stories describe the importance of emotional and financial support from the extended family to children who have been orphaned by HIV. One storyteller who had recently lost both parents and his grandmother to an unnamed illness explained the importance of social support in times of crisis: ‘The void of being left by my grandmother, closed because of the love we received at home from our family, who put us first before their own children. Love holds life together. Nothing can overcome love’ (man, aged 21). These words were accompanied by a drawing of a child surrounded by their family with a woman in the foreground carrying a pot of food. The words “Family, love and unity’ are inscribed above the people (see Fig 6 below).

Four of the stories highlight the negative consequences of poor social support. For example, in the story cited at the beginning of this article, the speaker explains how lack of social support led her mother to bury her ART pills (woman, aged 20). Another story explains how an HIV positive mother refused to take her pills and her young children were not able to help her without the support of the other adult family members: ‘If we had more family support, maybe mum would still be with us. At that time, our family distanced themselves from us. They did not want to hear anything’ (woman, aged 33). Although the theme of religious support does not emerge from a reading of story texts or artworks, it is suggested by song choices, with nine of the participants choosing to record Christian choral songs for their soundtracks (see Table 2).

## Discussion

Our primary goal in this paper was to better understand the nature of adherence to ART in an under-resourced, rural community of HIV high prevalence and to identify specific individual and structural factors that can either challenge or support adherence in this context. Through an inductive thematic analysis of twenty digital story texts, soundtracks and drawings six overlapping and nonexclusive themes emerged. The relatively broad selection of topics covered in the stories is indicative of the freedom that we gave participants to choose what information to share with us in the workshops. This strategy was motivated primarily by ethical considerations since we were aware that storytelling, particularly in this context, is a sensitive process and we did not want to coerce participants into sharing more than they were comfortable with. Moreover, since our interests as researchers lie in the personal contexts of ART adherence, it was beneficial to learn how adherence intersects with the everyday lives of people, rather than learning about it in isolation. Lastly, by collecting stories about adherence from the general community rather than from HIV positive people only, we were able to learn more about the broad context of adherence and how it is understood at a community level.

There were numerous references to other chronic diseases in these stories. This is a reminder of the rise of non-communicable diseases in South Africa and is suggestive that HIV needs to be understood in the context of other morbidities and co-morbidities [23]. The perception that HIV is like other chronic diseases is important since it suggests a growing belief in the efficacy of ART which is recognized as an essential facilitator to adherence and is suggestive of a growing confidence in HIV drugs [24]. A belief in HIV medication is echoed in the armed struggle subtheme, which alludes to the historical fight against Apartheid. Since Apartheid was overcome after many years of black resistance the reference to the resistance movement in this context can be seen to imply that in the context of ART, HIV can be overcome. However, the comparison between HIV and other chronic illness might not be such a positive sign for adherence. In fact, it might indicate that adherence could remain a challenge for this population, since it is well known that adherence to medication for other chronic illnesses is generally poor in this context [25; 26].

Interestingly while these stories suggest a growing confidence in ART, this is contrasted with an apparent decline in community belief in traditional/faith medicines, which are seen to delay HIV treatment, to be ineffective and to interfere with the use of ART. It is very likely that these accounts are influenced by a bias of social desirability, since participants would have been aware of our research centre’s support for Western medicines and ART. Nevertheless, these stories might also be seen to imply a growing awareness in the community that the ‘use of multiple health care modalities before ART initiation can lead to delayed HIV testing and ART initiation’ [27].

The tragic accounts of illness and death in these stories are very stirring and effectively move beyond the sterile statistics to paint a picture of the personal impact of HIV on human lives in South Africa. The three cases of MTCT, in particular the stories about infant deaths, are very sad and are symptomatic of an overburdened local healthcare system, which has sometimes failed to effectively deliver lifesaving PMTCT. It is important to note that the story about transmission through breastfeeding occurred in 1995 before the highly successful national PMTCT programme that was launched in 2001. Nevertheless, the inclusion of these stories, approximately 20 years after their occurrence, stresses the fact that past experiences may still have a profound impact and influence people’s beliefs, emotional wellbeing and health care behaviours.

It is clear from these accounts that fear of HIV testing is widespread and that rather than testing as a part of a regular healthcare check, people tend to test either when they are sick or fall pregnant. This could be seen as a barrier to accessing HIV treatment and care, since early linkage to care is important to the success of ART [28]. An analysis of participant drawings suggests that the journey to test for HIV is often, at least partially, made on foot. Colleagues have noted that ‘distances between health care settings and homes, as well as means to get between those are some of the impediments… for ART adherence’ [29]. One could further argue that the solitary figures in these drawings suggest that this journey is made alone, rather than in the company of a friend or family member. This could be acknowledged as a barrier to good adherence since social support is known to be of central importance [10].

The accounts of post-diagnosis depression and fear quoted above might be seen as further individual barriers to adherence [9]. Nonetheless, the reports of useful counselling and good medical advice from HIV lay counsellors at the clinics points to the valuable role that these workers play and the potential for positive relationships with healthcare workers to support good adherence [6]. Indeed, these stories would suggest that lay counsellors should be properly supported and valued within the framework of HIV care [30].

The themes of stigma, disclosure and family support are closely interwoven since a fear of stigma can have a major impact on a person’s ability to disclose and to receive the support that is key to successful adherence. These accounts suggest that there is still stigma associated with HIV infection. This is evident in the numerous accounts of dishonesty about HIV-related death and illness, of fear of testing and of individuals who failed to disclose their HIV status to loved ones. This finding is underlined by the recently published ‘People living with HIV stigma index’, which reported that ‘moderate levels of HIV-related external and internalized stigma and discrimination were found’ [31]. Stigma has been identified as one of the key impediments to the success of long-term treatment and these narratives provide personal evidence of how these individual barriers to ART adherence function in this rural society [2; 32].

It is evident that disclosing ones status to another can not only allow one to receive support and facilitate adherence, but can also inspire community change by encouraging others to test for HIV. It would seem from these stories that disclosure is often recommended by a lay counsellor and occurs most often between members of a family rather than between partners, where there is a risk of domestic violence and rejection. However, it is important to note that the drawings accompanying some of the stories make it clear that disclosing one’s status can be very difficult. Health interventions that encourage disclosure should ensure that appropriate psychological or social support is provided. Indeed, in other research, social support has been associated with good adherence [2; 6], and even in these stories it is evident that social support can play a key role in day-to-day survival, accessing medication, remembering to take medication and also emotional support. Furthermore, the predominance of religious songs in the sound tracks also suggests that individuals in this vulnerable community can find strength and consolation through their belief in Christianity.

Overall, we found digital stories to offer rich multimedia accounts of personal experience with multiple layers of meaning. However, the methodology did have certain limitations, which are outlined here. Firstly, the research would have been strengthened by in-depth interviews with participants to provide further insight into the significance and meaning of their stories. Secondly, there was a shortage of male participants; we suggest that future research projects use innovative recruiting techniques such as recruiting in workplace settings or at taxi ranks and *shebeens* (informal drinking taverns) to attempt to give further voice to male perceptions of adherence in this community. Thirdly, our focus was limited to medication adherence rather than broader concepts of adherence to treatment and care, although in the end much broader ideas about the lived experience of HIV and other illnesses came out in the process. Fourthly, the stories that were shared could have been recalled from any time; some stories might relate to the distant past and some to much more recent memories. This makes it somewhat difficult to identify the real, on going adherence challenges that are occurring right now. Lastly, generalizability could be a challenge since the local community has been exposed to Africa Centre’s health communication campaigns for many years and is probably more literate about HIV and adherence than in other areas.

## Conclusion

In conclusion, our inductive thematic analysis of twenty digital story texts, soundtracks and drawings provided pertinent insights into the nature of adherence in this resource poor, rural community and helped to identify potential barriers and facilitators for those on lifelong treatment. Many of the stories gathered in this study reflect a growing confidence in the effectiveness of ART at both the individual and the community level. This sense of agency and hope should be acknowledged as a key facilitator in successful adherence. The stories highlighted the complexity of the issues that individuals and households face as they deal with HIV and ART in this setting and suggest that ‘simple’ biomedical responses to improving adherence may have limited impact [33], Certainly, the narratives make it clear that HIV has taken a considerable toll on local communities and that an overburdened local healthcare system has often struggled to meet the needs and wants of a population suffering the consequences of a rapidly expanding HIV epidemic and to provide the necessary medical and emotional support. Moreover, poverty, evident in the lack of transport to seek medical care and in poor social support, can serve as a further structural barrier to accessing care and adhering well to medications. Lastly, it is important to note that although disclosure is a key step on the path to good adherence, the drawings that accompany some of the stories make it clear that disclosing one’s status can be very difficult. Health interventions that encourage disclosure should ensure that appropriate psycho-social support is provided.

Our analysis of the stories suggests several opportunities for further research and the design of novel health interventions to support optimal adherence. First, the acknowledgment by several storytellers that HIV is much like other chronic diseases suggests that we could use the knowledge accumulated through several years of research into ART adherence to design interventions that might improve adherence to other medications as well. Second, the clear link that emerged between social support and health-seeking behaviour suggests that future health promotion campaigns should encourage individuals to test together, or at least accompany each other for testing, to encourage social support from the outset. Additionally, perhaps, in this context, home-based testing and ART club interventions might be recommended to make it easier for individuals to adhere to their treatment regimes and to provide a sense of support and solidarity. Indeed, community based ART distribution has been implemented with success by Médecins Sans Frontières and other agencies to support ART expansion and retention in resource-limited settings in South Africa, Mozambique, Democratic Republic of Congo, Malawi and Zimbabwe [34; 35; 36; 37]. Perhaps, future health interventions in this community can also collaborate with local churches to engage community members and stimulate relevant social change.

## References

[1] Mills E, Nachega J, Buchan I, Orbinski J, Attaran A, Singh B, et al Adherence to antiretroviral therapy in sub-Saharan Africa and North America: a meta-analysis. JAMA. 2006; 296(6): 679–90. 16896111 10.1001/jama.296.6.679

[2] Peltzer K, Friend-du Preez N, Ramlagan S, Anderson J. Antiretroviral treatment adherence among HIV patients in KwaZulu-Natal, South Africa. BMC Public Health. 2010; 10: 111 10.1186/1471-2458-10-111 20205721 PMC2837855

[3] Mutevedzi P, Lessells R, Heller T, Bärnighausen T, Cooke G, Newell M-L. Scale-up of a decentralised HIV treatment programme in rural KwaZulu-Natal, South Africa: does rapid expansion affect patient outcomes? Bulletin of the World Health Organisation. 2010; 88: 593–600. 10.2471/BLT.09.069419 PMC2908968 20680124

[4] Manasa J, Lessells R, Skingsley A, Naidu K, Newell M-L, McGrath N, et al High-levels of acquired drug resistance in adult patients failing first-line antiretroviral therapy in a rural HIV treatment programme in KwaZulu-Natal, South Africa. PLoS One. 2013; 8(8): e72152 10.1371/journal.pone.0072152 23991055 PMC3749184

[5] Lessells R, Stott K, Manasa J, Naidu K, Skingsley A, Rossouw T, et al Implementing antiretroviral resistance testing in a primary health care HIV treatment programme in rural KwaZulu-Natal, South Africa: early experiences, achievements and challenges. BMC Health Services Research. 2014; 14:116 10.1186/1472-6963-14-116 24606875 PMC3973961

[6] Ross AJ, Aung M, Campbell L, Ogunbanjo GA. Factors that positively influence adherence to antiretroviral therapy by HIV and/or AIDS patients and their caregivers. African Journal Primary Health Care Family Medicine. 2011; 3(1): 196–199.

[7] Stirratt MJ, Remien RH, Smith A, Copeland O, Dolezal C, Krieger D, et al The Role of HIV Serostatus Disclosure in Antiretroviral Medication Adherence. AIDS and Behavior. 2006; 10 (5): 483–493. 16721505 10.1007/s10461-006-9106-6

[8] Michel J, Matlakala C, English R, Lessells R, Newell M-L. Collective patient behaviors derailing ART rollout in KwaZulu-Natal: perspectives of health care providers. AIDS Research and Therapy. 2013; 10: 20 10.1186/1742-6405-10-20 23870285 PMC3765690

[9] Grenard J, Munjas BA, Adams JL, Suttorp M, Maglione M, McGlynn EA, et al Depression and medication adherence in the treatment of chronic diseases in the United States: a meta-analysis. Journal General Internal Medicine. 2011; 26(10): 1175–82. 10.1007/s11606-011-1704-y PMC3181287 21533823

[10] Kagee A, Remien RH, Berkman A, Hoffman S, Campos L, Swartz L. Structural barriers to ART adherence in Southern Africa: challenges and potential ways forward. Global Public Health. 2011; 6(1): 83–97. 10.1080/17441691003796387 20509066 PMC3056922

[11] Chaiyachati K, Ogbuoji O, Price M, Suthar A, Negussie E, Bärnighausen T. Interventions to improve adherence to antiretroviral therapy: a global systematic review. AIDS. 2014; 28:S187–204. 10.1097/QAD.0000000000000252 24849479

[12] Bärnighausen T, Chaiyachati K, Chimbindi N, Peoples A, Haberer J, Newell MJ. Interventions to increase antiretroviral adherence in sub-Saharan Africa: a systematic review of evaluation studies. The Lancet Infectious Diseases. 2011; 11(12): 942–951. 10.1016/S1473-3099(11)70181-5 22030332 PMC4250825

[13] Lambert J. Digital Storytelling: Capturing lives creating community 3rd ed. Berkeley, California: Digital Diner Press; 2009.

[14] Cueva M, Kuhnley R, Revels L, Cueva K, Dignan M, Lanier A. Bridging storytelling traditions with digital technology. International Journal of Circumpolar Health. 2013; 72: 1–6. 10.3402/ijch.v72i0.20717 PMC3752288 23984267

[15] Zaidi J, Grapsa J, Tanser F, Newell M-L, Bärnighausen T. Dramatic increased in HIV prevalence after scale-up of antiretroviral treatment: a longitudinal population-based HIV surveillance study in rural KwaZulu-Natal. AIDS. 2013; 27: 2301–2305. 10.1097/QAD.0b013e328362e832 23669155 PMC4264533

[16] Houlihan C, Bland R, Mutevedzi P, Lessells R, Ndirangu J, Thulare H, et al Cohort profile: Hlabisa HIV Treatment and Care Programme’, *International* Journal Epidemiol. 2011; 40(2): 318–326. 10.1093/ije/dyp402 PMC3195268 20154009

[17] Bor J, Bärnighausen T, Newell C, Tanser F, Newell M-L. Social exposure to an antiretroviral treatment programme in rural KwaZulu-Natal. Tropical Medicine International Health. 2011; 16(8): 988–994. 10.1111/j.1365-3156.2011.02795.x 21615631 PMC4295018

[18] Bor J, Herbst A, Newell M-L, Bärnighausen T. Increase in adult life expectancy in rural South Africa: valuing the scale-up of HIV treatment. Science. 2013; (339): 961–965. 23430655 10.1126/science.1230413 PMC3860268

[19] Gubrium A. Digital storytelling: an emergent method for health promotion research and practice. Health Promotion Practice. 2009; 10(2): 186–91. 10.1177/1524839909332600 19372280

[20] Moletsane R, Mitchell C, de Lange N, Stuart J, Buthelezi T, Taylor M. What can a woman do with a camera? Turning the female gaze on poverty and HIV and AIDS in rural South Africa. International Journal of Qualitative Studies in Education. 2009; 22(3): 315–331.

[21] Riessman C. Narrative methods for the human sciences Newbury Park California: Sage; 2008.

[22] Riessman C. Narrative Analysis’ In: Lewis-Beck MS, Bryman A, Futing Liao T, editors. The Sage Encyclopaedia of Social Sciences Research Methods. Newbury Park, California (CA): Sage; 2004 pp 705–09.

[23] Mayosi BM, Fisher AJ, Lalloo UG, Sitas F, Tollman SM, Bradshaw D. The burden of non-communicable diseases in South Africa. Lancet. 2009; 374: 934–947. 10.1016/S0140-6736(09)61087-4 19709736

[24] Ross AJ, Aung M, Campbell L, Ogunbanjo GA. Factors that positively influence adherence to antiretroviral therapy by HIV and/or AIDS patients and their caregivers. African Journal Primary Health Care Family Medicine. 2011; 3(1): 196–199.

[25] Krass I, Schieback P, Dhippayom T. Adherence to diabetes medication: a systematic review. Diabetic Medicine. 2015; 32(6): 725–737. 10.1111/dme.12651 25440507

[26] Schroeder K, Fahey T, Ebrahim S. How can we improve adherence to blood pressure-lowering medication in ambulatory care? Systematic review of randomized controlled trials. JAMA Internal Medicine. 2004; 164(7): 722–732 10.1001/archinte.164.7.722 15078641

[27] Moshabela M, Pronyk P, Williams H, Schneider H and Lurie M. Patterns and implications of medical pluralism among HIV/AIDS patients in rural South Africa. AIDS Behaviour. 2015; 15(4): 842–52. 10.1007/s10461-010-9747-3 PMC4790116 20628898

[28] Mugavero M, Amico KR, Westfall AO, Crane H, Zinski A, Willig JH, et al Early retention in HIV care and viral load suppression: implications for a test and treat approach to HIV prevention. Journal of Acquired Immune Deficiency Syndromes. 2012; 59(1): 86–93. 10.1097/QAI.0b013e318236f7d2 21937921 PMC3237801

[29] Bam K, Rajbhandari RM, Karmacharya DB, Dixit SM. Strengthening adherence to Anti Retroviral Therapy (ART) monitoring and support: operation research to identify barriers and facilitators in Nepal. Bio Medical Central (BMC) Health Services Research. 2015; 15: 188. 10.1186/s12913-015-0846-8 PMC4428010 25939593

[30] Petersen I, Fairall L, Egbe CO, Bhana A. Optimizing lay counsellor services for chronic care in South Africa: a qualitative systematic review. Patient Education & Counselling. 2014; 95(2): 201–210. 10.1016/j.pec.2014.02.001 24629835

[31] South African National AIDS Council (SANAC). The People Living With HIV Stigma Index: South Africa 2014—Summary Report. [Internet]. 2014 [cited 2015 Oct 19]. Available from: <http://www.sanac.org.za/resources/cat_view/7-publications/9-reports>,

[32] Katz IT, Ryu AE, Onuegbu AG, Psaros C, Weiser SD, Bangsber DR, et al Impact of HIV-related stigma on treatment adherence: systematic review and meta-synthesis. Journal of the International AIDS Society. 2013; 16 (3Suppl 2): 18640 10.7448/IAS.16.3.18640 24242258 PMC3833107

[33] Doyal L. The art of medicine. A decade of researching the social aspects of HIV and AIDS. Lancet. 2014; 384: 2012–2013. 10.1016/s0140-6736(14)62362-x 25517004

[34] Luque-Fernandez MA, Van Cutsem G, Goemaere E, Hilderbrand K, Schomaker M, Mantangana N. Effectiveness of Patient Adherence Groups as a Model of Care for Stable Patients on Antiretroviral Therapy in Khayelitsha, Cape Town, South Africa. PLoS ONE. 2013; 8(2): e56088 10.1371/journal.pone.0056088 23418518 PMC3571960

[35] Wilkinson L. ART adherence clubs: A long-term retention strategy for clinically stable patients receiving antiretroviral therapy. Southern African Journal of HIV Medicine [Internet]. 2013 [cited 2015 March 25]; 14: Available from: <http://www.sajhivmed.org.za/index.php/hivmed/article/view/77/118>, accessed 5 August 2015.

[36] Grimsrud A. Sharp J, Kalombo C, Bekker LG, Myer L. Implementation of community-based adherence clubs for stable antiretroviral therapy patients in Cape Town, South Africa. Journal of the International AIDS Society. 2015; 18:19984 10.7448/IAS.18.1.19984 26022654 PMC4444752

[37] Bemelmans M, Baert S, Goemaere E, Wilkinson L, Vandendyck M, van Cutsem G, et al Community-supported models of care for people on HIV treatment in sub-Saharan Africa. Tropical Medicine International Health. 2014; 19(8): 968–77. 10.1111/tmi.12332 24889337

